# Development and implementation of a community-based research network

**DOI:** 10.1017/cts.2020.45

**Published:** 2020-05-18

**Authors:** Brittany C. Minor, Jessica Dashner, Sandra M. Espín Tello, Rebecca Bollinger, Marian Keglovits, James Stowe, Margaret Campbell, Susan L. Stark

**Affiliations:** 1 Program in Occupational Therapy, Washington University in St. Louis, St. Louis, MO, USA; 2 Department of Neurology, Washington University in St. Louis, St. Louis, MO, USA; 3 Department of Informatics, University of the Basque Country (UPV/EHU), Leioa, Spain; 4 Mid-America Regional Council, Kansas City, MO, USA; 5 Campbell & Associates Consulting, Grapeview, WA, USA

**Keywords:** Community partnerships, aging, disability, PBRN, community-based

## Abstract

**Introduction:**

People aging with long-term physical disabilities (PAwLTPD), meaning individuals with onset of disability from birth through midlife, often require long-term support services (LTSS) to remain independence. The LTSS system is fragmented into aging and disability organizations with little communication between them. In addition, there are currently no evidence-based LTSS-type programs listed on the Administration for Community Living website that have been demonstrated to be effective for PAwLTPD. Because of these gaps, we have developed a community-based research network (CBRN), drawing on the practice-based research network model (PBRN), to bring together aging and disability organizations to address the lack of evidence-based programs for PAwLTPD.

**Materials and Methods:**

Community-based organizations serving PAwLTPD across the state of Missouri were recruited to join the CBRN. A formative process evaluation of the network was conducted after a year to evaluate the effectiveness of the network.

**Results:**

Nine community-based organizations across the state of Missouri joined the CBRN. CBRN members include three centers for independent living (CILs), three area agencies on aging (AAAs), one CIL/AAA hybrid, one non-CIL disability organization, and one non-AAA aging organization. To date, we have held seven meetings, provided educational opportunities for CBRN members, and launched an inaugural research study within the CBRN. Formative evaluation data indicate that CBRN members feel that participation in the CBRN is beneficial.

**Conclusion:**

The PBRN model appears to be a feasible framework for use with community-based organizations to facilitate communication between agencies and to support research aimed at addressing the needs of PAwLTPD.

## Introduction

In the United States, 15.4 million adults are aging with a disability [1]. People aging with long-term physical disabilities (PAwLTPD) often experience the onset of secondary conditions such as pain [2–4], weakness [5], and fatigue [5,6] as a result of their primary disabling condition, as well as early onset of age-related health conditions, leading to decreased function [7–9]. Participation or “involvement in a life situation” such as work, school, play, social, civic, or religious activities is also diminished for many individuals aging with disability [10]. Decreased function and participation have consequences, including poor quality of life [11] and negative health outcomes [8].

Long-term support services (LTSS) can reduce functional and participation limitations. Two common LTSS providers are Centers for Independent Living (CILs) and Area Agencies on Aging (AAAs). CILs and AAAs are community-based organizations that provide services for adults with disabilities and older adults. Together, they represent approximately 1000 agencies across the United States [12,13]. Traditionally, CILs and AAAs have focused on services to facilitate long-term care or independent living, but not both. For example, CILs primarily focus on independent living issues for people with disabilities such as information and referral; independent living skills training, and peer counseling [14]; rather than issues pertaining to long-term care. In contrast, AAAs provide LTSS with a focus on long-term care, such as home-delivered meals, or homemaker assistance but rarely focus on independent living issues. Together CILs and AAAs provide a variety of services to PAwLTPD including, but not limited to, personal care attendants, health and wellness classes, support groups, home repairs, meal services, case management, and transportation. Although both agencies serve PAwLTPD, services often do not focus on the unique needs of PAwLTPD.

CILs and AAAs are encouraged, and in some cases, federally mandated [15] to use evidence-based (EB) interventions. EB interventions, such as Tai Chi: Moving for Better Balance [16], are beneficial for people aging without a disability or with Parkinson’s disease, but it is unknown if it is effective for other PAwLTPD. PAwLTPD have a wide range of functional impairments and multiple comorbidities that vary by disability and often exclude them from participating in clinical trials [17–19]. The exclusion of PAwLTPD from most clinical trials limits the applicability of evidence-based programs for this target population. None have been tested, or even demonstrated effectiveness in facilitating independence and community participation for PAwLTPD. Additionally, translation of evidence from gerontological-based interventions focused on the health, wellness and community participation of older adults to PAwLTPD is sorely lacking [20,21].

The Administration on Community Living (ACL) within the US Department of Health and Human Services places a strong emphasis on developing and funding EB practices among AAAs. The ACL is also home to the National Institute on Disability, Independent Living, and Rehabilitation Research (NIDILRR), which funds research to promote the effective use of evidence “to improve the abilities of people with disabilities to perform activities of their choice in the community” [22]. ACL has published criteria for defining EB practice; yet, to date, few interventions have met these criteria [15,23]. For community-based organizations to meet the needs of PAwLTPD, they need EB programs tailored to this growing demographic group. There is an urgent need for rigorously tested programs to improve community participation for PAwLTPD.

Practice-based research networks (PBRNs) provide a model for bringing together researchers and service providers to develop EB programs [24]. PBRNs are networks of community-based primary care practices and researchers who work together to fill gaps in service with EB interventions [25]. PBRNs quickly identify, address, and implement solutions to real-world problems that matter to their communities and use their findings to update network members’ practices [26]. PBRN priorities are driven by the needs of the community service providers. It can take almost 17 years for new research findings developed in isolation in a laboratory to be implemented in practice, and many interventions do not make it to implementation because of a failure to understand the context in which the intervention would be delivered [27,28]. PBRNs were created to reduce this translation gap to improve care [24,29].

To reduce the translation gap of interventions for PAwLTPD, we adapted the PBRN approach for community-based organizations and created a new community-based research network (CBRN) designed to support translational research to serve PAwLTPD. The aim of this study was to determine if a PBRN model is feasible as a framework for use with community-based long-term support service organizations.

## Materials and Methods

All study procedures were reviewed and approved by the Washington University in St. Louis Human Research Protection Office. We followed the PBRN model to build and then evaluate a CBRN. We are continuing to modify our procedures to meet the needs of the participating organizations.

## Participants

Potential CBRN members were recruited via direct contact, phone or email, or self-referral. The inclusion criteria were: (a) community-based organization, (b) provide services to PAwLTPD, (c) part of a national organization, and (d) located within the state of Missouri. There were no exclusion criteria.

## Formation of CBRN

Practice-Based Research Networks (PBRN) are networks of primary-care medical practices that have formed to address quality improvement, and practice change [24]. We used the PBRN model to build a CBRN infrastructure to support: (1) recruiting and retaining of members, (2) defining a clear mission and vision, (3) developing an organizational structure, (4) providing expertise to support the mission of the organization, and (5) building infrastructural capacity for long-term sustainability. We recruited community-based organizations that expressed interest in participating in the CBRN by inviting them to join the CBRN. We then surveyed them to identify a date for an initial meeting, the best methods of communication, current issues faced while serving PAwLTPD, and current services provided. Quarterly meetings were held to build rapport among members, define a name and a clear mission for the network, develop organizational structure for the network, consult with experts in aging and disability research who could provide expertise to support the mission, and design the inaugural research study. CBRN members were provided $50/hour as payment for participation in the CBRN, this includes meeting attendance, preparation for meetings, and time spent working on the design of the inaugural research study. All CBRN members were surveyed after 1 year to evaluate the progress.

## Measures

The organizations were surveyed to describe their services and participants, determine preferences for meeting times and format, and identify preliminary issues facing their agencies.

The Practice-Based Research Network (PBRN) Research Good Practices recommend yearly review of network goals to determine progress [30]. To measure the effectiveness of the network’s operating procedures and to review network goals, we adapted the Partnership Self-Assessment Tool – Questionnaire [31] to create a formative evaluation of the network. The Partnership Self-Assessment Tool – Questionnaire measures a partnership’s synergy, or its ability to combine the knowledge, skills, and resources of each member of the partnership to accomplish more than any individual member could do on its own [32,33]. The tool also identifies the strengths and weaknesses of a partnership that are related to synergy: leadership; administration and management; efficiency; availability of resources; and members’ perspectives on the decision-making process, benefits and drawbacks of participation, and overall satisfaction. The tool is intended for use by partnerships that are at least 6 months old, have at least five active members, have begun to take action to implement their plans, and are continually working together to develop and modify their goals and plans [33].

We used 52 items from the scale to assess the following components: (1) identifying our strengths and weaknesses in terms of leadership, (2) availability of network resources, (3) members’ perspectives on the decision-making process, and (4) benefits and drawbacks of participation. Leadership items were measured on a 4-point scale with response categories ranging from *very good* to *poor*, with an option to choose *don’t know*. Network resource items were measured on a 4-point scale with response categories ranging from *all of what it needs* to *none of what it needs*, with an option to choose *don’t know*. Items related to decision-making were measured on a 4-point scale with response categories ranging from *extremely comfortable* to *not at all comfortable,* and *all of the time* to *none of the time*, with an option to choose *don’t know*. Benefit items were measured on a 4-point scale with response categories ranging from *very good potential* to *poor potential*, with an option to choose *don’t know*. CBRN members were asked to answer *yes* or *no* questions related to drawbacks.

In addition, questions related to the importance of goals were measured on a 4-point scale with response categories ranging from *extremely important* to *not at all important* or *don’t know*. Items related to achieving goals were measured on a 4-point scale with response categories ranging from *very well* to *not at all*, with an option to choose *don’t know*. Questions related to how supported CBRN members felt participating in the network were measured on a 4-point scale with response categories ranging from *very supported* to *not at all supported*, with an option to choose *don’t know*. Barriers to serving PAwLTPD, identifying services needed for PAwLTPD, barriers to participation in the partnership, efficiency gaps, and ways that the partnership could better benefit individual partners were measured with qualitative questions.

We asked one representative from each of our active CBRN member organizations to complete the survey, as well as several members of the research staff. All CBRN member organizations who attended at least two quarterly meetings were considered active members. A convenience sample of research staff who had attended at least two quarterly meetings were asked to participate. Survey responses were returned anonymously. Data for both the initial survey and the formative evaluation were collected and managed using the Research Electronic Data Capture (REDCap) system. REDCap [34] is a secure, web-based application designed to support data capture for research studies.

## Data Analysis

Data from the initial survey and the closed-ended questions from the formative evaluation are presented as frequencies. Thematic analysis of survey responses was conducted by a research assistant and two of the paper authors (MK and BM) with training and experience in qualitative research methods. Survey responses were de-identified prior to storage and content coding was completed in NVivo version 12.0. A grounded theory approach and constant comparison were used to identify themes emerging after open and axial coding of survey responses to identify consistencies and differences among coded terms [35–38]. Themes were validated with the CBRN members for member checking to enhance the trustworthiness of qualitative findings [39,40].

## Results

### Formation of CBRN

**Fig. 1.**
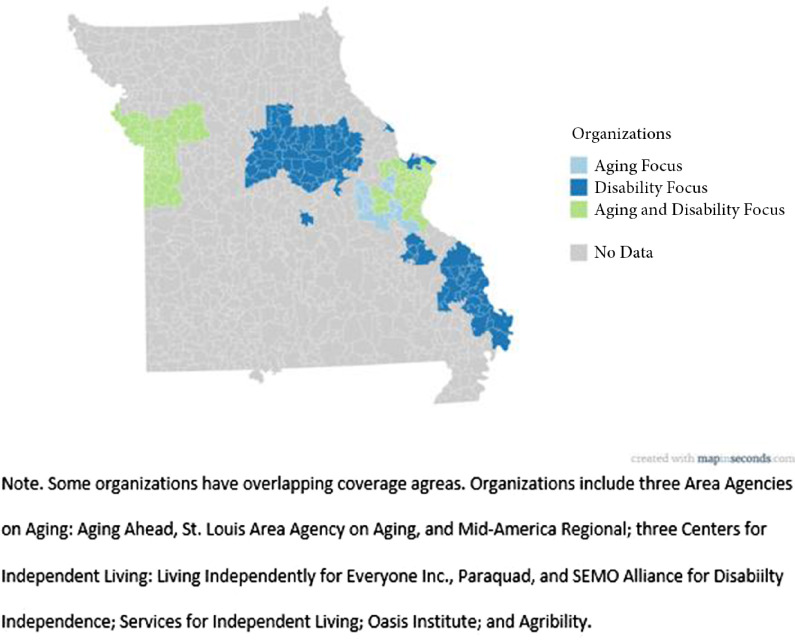
Geographic coverage of the community-based research network (CBRN).

We successfully recruited nine community-based organizations across the state of Missouri to join the CBRN. Between September and December 2017, a convenience sample of eight community-based organizations volunteered to participate in the CBRN. Seven members were organizations that had previously worked with the research staff on other projects. One organization agreed to participate after our presentation at the Missouri Summit on Aging and Health. The last organization expressed interest in participating after hearing about our study from other organizations. The CBRN members include three CILs, three AAAs, one CIL/AAA hybrid, one non-CIL disability organization, and one non-AAA aging organization (Fig. 1).

The CBRN has been officially named the Missouri Aging and Disability Research Network. We have established a mission to accelerate the translation, adaptation, evaluation and availability of evidence-based interventions designed to improve independence and community participation for people aging with long-term physical disabilities (PAwLTPD). The goals of the CBRN are to:

1. develop interventions for PAwLTPD;
2. help people learn about evidence-based practice delivered in the community;
3. understand the bridge between disability and aging services;
4. be part of a coalition of providers that bridge across aging and disability; and
5. link with researchers, agencies and participants to share knowledge.

The CBRN holds four quarterly meetings each year where we discuss current research projects being conducted or planned in the CBRN, upcoming educational opportunities, upcoming events being held by member organizations, resources available to organizations and to PAwLTPD and plans for future meetings.

Our initial activities within the CBRN focused on goals 3–4. To date, we have held seven meetings, including one in-person meeting. We have an average of 10 attendees per meeting. Meetings continue to be held via teleconference for the convenience of all members. To address our second goal of helping people learn about evidence-based practice delivered in the community, we have provided educational opportunities for CBRN members and the community. This includes three webinars on the topics of “Aging, Disability, and Depression,” “Technology for Addressing Toileting for People Aging with Disabilities,” and “Fall Prevention.” The webinars were attended live and are available online. We have had over 50 views of our webinars to date. To address our fifth goal to link with researchers, agencies and participants to share knowledge, we have presented on the topic of developing and implementing a CBRN at two different stakeholder conferences: a representative from the St. Louis Area Agency on Aging and a CBRN investigator co-presented at the National Association of Area Agencies on Aging conference in 2019, and the CBRN research team also presented at the 2018 National Association of Research and Training Centers (NARRTC) conference in Washington, DC.

To begin to address the CBRN’s first goal, we launched our first study within the CBRN in 2017. The initial study is a longitudinal cohort study of PAwLTPD aged 45–64. We are surveying participants to examine changes in health and community participation over 3 years, as well as personal and environmental factors that facilitate and impede both. The CBRN has been our primary recruitment source for this study. We worked with the CBRN members to employ several recruitment methods. These included in-person recruitment at community events held by the CBRN members, such as health fairs and bus pass distribution meetings, as well as sending out flyers with CBRN member organization newsletters, as separate mailings, and with home-delivered meals. In addition, some CBRN members provided call lists for eligible participants, which allowed us to speak directly to participants about the study and answer any questions prior to enrollment. We also utilized social media for recruitment. This included creating a website and a Facebook page for the CBRN, where we post updates about the survey and run ads for study participants. Members of the CBRN also were able to share posts to their organization pages and create their own posts as part of the recruitment effort.

### Formative Evaluation Results

#### Evaluation of the network

Twelve representatives of the CBRN completed the formative evaluation of the network (Table 1). This included seven representatives from the active CBRN organizations and five research staff. Scores ranged from 2.8 to 3.7 on a 4-point scale for items related to the goals of the CBRN, strengths and weaknesses of leadership, availability of network resources, benefits of participation, and decision making. All of the items concerning drawbacks had at least one respondent who reported experiencing a drawback, except for the item “receiving insufficient credit for participation or accomplishments.” Respondents were most likely to report that participation in the network “takes too much time and resources away from primary duties,” with 25% of respondents indicating such. Despite this, all respondents reported that the benefits of participation exceeded the drawbacks. All respondents also reported feeling *somewhat supported* to *very supported* in their participation in the network.

**Table 1.** Formative evaluation results

Category	*n* = 12
Importance of CBRN goals to your organization’s mission	3.8
Progress in achieving CBRN goals	3.4
Identifying strengths and weaknesses of leadership	3.2
Availability of network resources	2.8
Potential benefits of participation	3.4
Perceptions of the decision making process	3.7

Average scores for responses in each category are shown; scores ranged from 0 to 4 with a higher score indicating more positive feedback. CBRN, community-based research network.

#### Formative evaluation qualitative responses

The response rate for the open-ended questions ranged from 42% to 92% (Table 2). One respondent replied *N/A* to all open-ended questions, so the responses were not included. The open-ended questions were divided into two major categories: serving PAwLTPD and participation in the CBRN (Table 3). All respondents were allowed to contribute answers. Research staff included five occupational therapists with experience working with people with disabilities, two of whom have physical disabilities. In terms of serving PAwLTPD, respondents’ primary concerns were marketing to obtain new clients who are PAwLTPD and providing services that are easy to access as PAwLTPD notice changes in their health and function. The services perceived as most important for PAwLTPD were social support and transportation. In terms of participation in the CBRN, respondents cited conflicting schedules and in-person meetings as the primary barriers to participation. The primary facilitators of participation in the CBRN were remote meetings and provision of useful resources.

**Table 2.** Barriers, challenges, and most-needed services

		Frequency of responses, *n* = 12
Challenges to serving PAwLTPD	Providing services that are easy to access when and where they are needed	10
	Marketing to obtain new clients who are PAwLTPD	7
	Providing services that PAwLTPD perceive are strongly needed	4
	Providing services with evidence of effectiveness for PAwLTPD	4
Services perceived as most important for PAwLTPD	Social support	10
	Transportation	8
	Personal safety	3
	Accessible housing	3
	Health care and medical equipment	3
	Financial assistance	2
	Exercise activities	1
Barriers to participation in community-based research network (CBRN) activities	Meeting times conflict with individual schedules	7
	Meetings conducted in person	2
	Activities and resources do not match organization’s needs	1
	Reduced clarity of remote communication methods (eg, phone)	1

There are more responses than the total number of respondents. Respondents were able to provide multiple examples. PAwLTPD, people aging with long-term physical disabilities.

**Table 3.** Examples of survey responses

Challenges to serving PAwLTPD	The main issue is that it’s not client focused, flexible, on-demand services. That takes lots of [money] to effectively provide.
	Access to services as they notice changes in function, even if they haven’t had a medical event.
	Lack of financial resources.
	Staying connected to the population.
	Lack of [evidence-based] interventions.
Services perceived as most important for PAwLTPD	[…]support network – having people able to help in a crisis, that will be dependable & HELP.
	Access to more financial resources (utilities paid, healthy foods, *money to socialize*).
	Reliable assistance when they need it.
	There are several transportation options but I’ve heard anecdotally that sometimes it’s difficult to find, good, consistent reliable help. And even the transportation options aren’t without drawbacks.
Barriers to participation in community-based research network (CBRN) activities	Only barrier I can think of is time.
	Other work obligations.
	Sometimes just other time commitments.
Facilitators of participation in CBRN activities	Conference calls are much easier than in person meetings.
	Having the early meetings makes easier to attend the conference calls.
	Webinars are very informative.

## Discussion

Our primary finding is that it is possible to establish and implement a cross-stakeholder aging and disability CBRN to better meet the needs of PAwLTD using the PBRN model. Our results are consistent with other studies that have used the PBRN model to form their own primary-care based PBRNs [26]. Our network varied in that we had a smaller recruitment pool than a network looking to recruit pediatric medical practices. We were able to establish CBRN membership by leveraging our social capital to gain support from community-based organizations. We were fortunate to be able to build on a history of successful partnerships with many of the community-based organizations to recruit them into the network. We used examples of our successful partnerships to recruit additional members.

Another key factor in establishing membership is that we were able to identify a unifying topic to bring together CILs and AAAs; namely, the shared need to improve community participation among individuals aging with long-term physical disabilities. Although we have been successful in collaborating with individual CILs and AAAs around this topic, we have experienced barriers when attempting to collaborate with local CILs and AAAs simultaneously. Addressing the needs of PAwLTPD is an important enough issue to inspire these organizations to come together. In both the initial survey and the formative evaluation, all of the CBRN members reported difficulties serving people aged 50–64 with physical disabilities. CBRN members struggle to serve PAwLTPD because the population can be difficult to reach and may be socially isolated. CBRN members also reported being limited by funding resources and a lack of EB programming for this population. Both groups have a strong interest in finding ways to overcome these difficulties for this population.

PAwLTPD are a unique, but growing, population that is often overlooked by researchers and policy makers [19]. They are part of a diverse segment of middle-aged individuals that is frequently excluded from both national surveys and clinical and behavioral intervention research. Moreover, as they grow older, they will age into a group of older adults with significant impairments and disabling conditions that has also historically been excluded from research [19,21]. The problem is that there are few EB interventions designed for implementation in community settings that have been developed or validated for either middle-aged or older adults with disabilities. Because of this gap in the availability of EB programs for people aging with long-term physical disabilities, the successful formation of a CBRN to address their needs has the potential to be very impactful. The initial cohort study aims to identify areas of need where we may be able to begin developing EB interventions for PAwLTPD served by our CBRN. However, we have not yet begun to address this issue directly.

Taken together, responses from the formative evaluation indicate that our CBRN members feel that this new cross-agency network is already achieving its goals and, at the same time, has room to grow. In general, members feel that the current goals of the CBRN are important to their organizations’ missions and that we are making progress toward achieving our goals. While all of our members reported that there are drawbacks to participation in the CBRN, they also indicated that the drawbacks were outweighed by the benefits of participation. Members also felt supported by their organizations to continue participation.

One limitation of forming a CBRN is that participation in the CBRN adds increased work for our members. The primary drawback of participation reported by members was the amount of time required for participation in the CBRN. Most of our members are dealing with budget cuts and decreased staffing in their organizations; it is important that the demands of the CBRN do not overwhelm them. At the same time, we want to empower them to participate in the CBRN and to feel that they can shape the future of the network. One way of doing that is to provide members with the tools they need to develop their own research questions via educational opportunities. Another way of maintaining engagement is to recognize the time and effort it takes to participate in the CBRN and to compensate members fairly for this time. We are fortunate to have funding for this currently, but we will need to be vigilant about maintaining funding for the CBRN to continue this practice.

Another limitation of this CBRN is our somewhat narrow focus on PAwLTPD. CILs and AAAs serve larger populations that include a much wider age range than 45–65 years, and people with and without disabilities. This could potentially cause a conflict for our members and was one of the barriers reported in our formative evaluation. One member reported, “The population focus of [the CBRN] is too specific for our generalist mandate; heavily competing obligations.”

Going forward, our next challenge will be ensuring that CBRN members feel empowered and have the tools necessary to collaborate to develop and investigate research questions, as well as assist in the translation and implementation of new EB practices to address those questions and improve community participation outcomes for individuals aging with long-term physical disabilities. We also plan to recruit more academic partners interested in conducting community-engaged research investigating questions presented by CBRN members.

Another challenge for us will be in taking the final step in building CBRN infrastructure by ensuring the long-term sustainability of the network. We will need to identify sources of support to maintain the network. As we develop more research questions, we may be able to apply for funding to address those questions. However, we will also need funding to sustain the network as it works to identify new questions.

## Conclusion

We have taken the first steps in closing the gap in communication between community agencies and researchers serving PAwLTPD. We have identified a topic that has relevance to unite two types of community agencies that have historically not worked together. We have also found that community-based agencies are interested in engaging in research that will benefit their clients and further the mission of their organizations. The CBRN shows promise as an effective instrument of collaborative research partnerships between researchers and community agencies to improve the health and participation of PAwLTPD.
